# Salinity Effects on Aquatic and Host Intestinal Microbiota Dynamics in *Rhinogobio ventralis*

**DOI:** 10.3390/ani15233407

**Published:** 2025-11-26

**Authors:** Kaixuan Liu, Qiang Zhao, Tianzhi Jin, Xuemei Li, Hanchang Sun, Xingbing Wu, Hailong Ge, Fang Li

**Affiliations:** 1 Integrative Science Center of Germplasm Creation in Western China (Chongqing) Science City, MOE Key Laboratory of Freshwater Fish Reproduction and Development, College of Fisheries, Southwest University, Chongqing 400715, China; 18359461500@163.com (K.L.); luckzhao0823@163.com (Q.Z.); 1120200513@mail.nankai.edu.cn (T.J.); 2 Key Laboratory of Freshwater Biodiversity Conservation, Ministry of Agriculture and Rural Affairs, Yangtze River Fisheries Research Institute, Chinese Academy of Fishery Sciences, Wuhan 430223, China; xmli@yfi.ac.cn (X.L.); wxbinq@yfi.ac.cn (X.W.); 3 College of Smart Agriculture, Technology Innovation Center of Ecological Fishery Industrialization, Chongqing University of Arts and Sciences, Chongqing 402160, China; sunhanchang199@163.com; 4 Postdoctoral Research Workstation of Chongqing Fengdu Agricultural Science and Technology Development Group Co., Ltd., Chongqing 408299, China

**Keywords:** salinity, *Ichthyophthirius multifiliis*, intestinal microbiome, environmental microbial communities, *Rhinogobio ventralis*, ecological trade-off

## Abstract

The endangered *Rhinogobio ventralis*, endemic to the upper Yangtze River, is dependent on captive breeding for its conservation. However, this highly stress-sensitive species is exceptionally susceptible to *Ichthyophthirius multifiliis*, leading to severe pathology and high mortality in culture. Elevated salinity holds potential for managing key aquaculture pathogens, including *Ichthyophthirius multifiliis* and *Saprolegnia* spp. However, its potential unintended ecological consequences remain insufficiently understood. This study evaluated the systemic impacts of 5‰ salinity on the culture environment of the endangered species *Rhinogobio ventralis*, using integrated 16S/18S rRNA gene sequencing and water quality analysis. The results demonstrated that while salinity treatment effectively reduced the environmental molecular signal of harmful eukaryotes such as *Ichthyophthirius* and *Saprolegnia*, it also induced significant ecological shifts: (1) aquatic prokaryotic diversity increased, yet the self-purification capacity of the water was compromised, indicated by elevated dissolved oxygen, nitrate nitrogen, and total nitrogen; (2) in the fish intestinal microbiome, a decline in potentially beneficial taxa (e.g., *Exiguobacterium*) co-occurred with an enrichment of genera containing potentially pathogenic species (e.g., *Staphylococcus* and *Pseudomonas*), collectively suggesting a state of dysbiosis; (3) co-occurrence network analysis revealed that the aquatic microbial community developed greater complexity, while the intestinal network became structurally simplified and more antagonistic. These findings reveal that elevating salinity in freshwater aquaculture systems compromises both host microbiome health and aquatic ecosystem functioning. As such, future aquaculture management should integrate supportive measures like probiotic supplementation to maintain overall system stability.

## Simple Summary

The endangered fish *Rhinogobio ventralis* is highly susceptible to “white spot disease” caused by *Ichthyophthirius multifiliis*, making artificial cultivation challenging. Although studies have shown that adding salt can help control such parasites, its effects on the aquatic environment and the fish intestinal microbiota remain unclear. This study evaluated the effects of low-concentration salt (5‰) in freshwater aquaculture by monitoring water quality and microbial communities in both the aquatic environment and the fish intestine. The results showed that while salt treatment effectively suppressed harmful organisms such as *I. multifiliis* and *Saprolegnia* in the water, it also induced a series of negative effects. The self-purification capacity of the water declined, manifested as significant increases in dissolved oxygen and nitrogen levels, and the balance of the fish intestinal microbiota was disrupted, characterized by a significant increase in microbial diversity, enrichment of potentially pathogenic bacteria (e.g., *Staphylococcus* and *Pseudomonas*), and a decrease in the relative abundance of the beneficial bacterium *Exiguobacterium*. Our findings reveal that employing salinity for parasite control compromises the self-purification capacity of aquatic systems and disrupts intestinal microbial community stability. We therefore propose integrating salinity management with probiotic supplementation to establish truly sustainable aquaculture systems.

## 1. Introduction

*Rhinogobio ventralis*, a species belonging to the order Cypriniformes, family Cyprinidae, and subfamily Gobioninae, is an endemic fish in the upper reaches of the Yangtze River in China. Valued for its nutritional quality and economic importance [1]. However, its natural populations are critically endangered, having been classified as “Endangered” in the China Red List of Vertebrates in 2016 [2] and listed as a National Grade II Protected Wildlife Species in 2021. Artificial breeding is crucial for its conservation. However, this species is highly stress-sensitive and susceptible to disease in culture, especially Ichthyophthirius multifiliis. A recent study of *R. ventralis* demonstrated that *I. multifiliis* infection causes severe skin pathology, immune dysregulation, and metabolic disruption, underlying the high mortality rates [3]. However, no effective treatment is currently available [4,5]. Frequent outbreaks of *I. multifiliis* infections necessitate energy-intensive closed recirculating aquaculture systems (RAS) with high filtration and disinfection requirements, significantly impeding large-scale breeding efforts. Therefore, developing energy-efficient methods to control *I. multifiliis* is essential for conserving *R. ventralis* germplasm resources.

Studies indicate that elevated water salinity effectively mitigates *I. multifiliis* infections in fish. Ward [6] demonstrated that *Gila robusta* cohabitated with *I. multifiliis*-infected *Agosia chrysogaster* in 3‰ salinity water remained healthy, while all individuals in freshwater died within 8 days. In vitro experiments demonstrated that salinities above 5‰ inhibit the development and hatching of the parasite’s infective stages [7]. The growth of the oomycete S. parasitica is also inhibited by increased salinity [8], reinforcing the broad inhibitory role of salt against aquatic pathogens. Additionally, studies confirm the safety of moderate salinity in freshwater aquaculture. Bal et al. [9] reported no notable alterations in specific growth rate, hematological parameters, or feeding behavior in *Heteropneustes fossilis* reared at 3‰ salinity for 45 days. Furthermore, Jia et al. [10] confirmed that grass carp (*Ctenopharyngodon idella*) maintained normal growth performance under 5‰ salinity during a continuous 42-day exposure. These findings collectively indicate that controlled salinity strategies are associated with a reduction in *I. multifiliis* infection signals while generally causing no significant physiological harm to fish, suggesting this approach holds promise as a foundation for energy-efficient solutions for *I. multifiliis* management in *R. ventralis* aquaculture. However, the ecological cost of elevated salinity for host-associated microbial communities remains unassessed.

As a critical environmental filter and osmotic stressor, salinity exerts a profound influence on microbial communities [11]. It restructures microbial communities and their metabolic functions in the water [12,13]. Salinity also indirectly shapes the intestinal microbiota of fish by altering both the environmental microbial pool and host-derived nutrient niches [12,14]. Consequently, the evaluation of any salinity intervention must account for its broad, systemic consequences rather than a narrow focus on a single pathogen. Despite its use in aquaculture, a holistic understanding of how salinity shapes the microbial ecology of both the water and the host intestine in conservation-oriented systems is lacking.

This study investigates how 5‰ salinity affects the aquatic microbiota, intestinal microbial ecosystem, and eukaryotic communities of the endangered *R. ventralis*. We focus specifically on its under-explored consequences for host-microbe homeostasis and overall microecological balance. Using integrated 16S/18S rRNA sequencing and water quality analysis, we aimed to (1) characterize structural and compositional shifts in both the aquatic microbiota and fish intestinal microbiota under 5‰ salinity exposure; (2) compare salinity-driven changes in microbial interaction networks between the aquatic environment and the host intestine; and (3) evaluate the ecological safety of using elevated salinity to control aquatic eukaryotic pathogens in conservation-oriented aquaculture.

## 2. Materials and Methods

### 2.1. Ethical Statement

All animal procedures in this research were conducted in accordance with the Guidelines for the Care and Use of Laboratory Animals in China and approved by the Southwest University Animal Ethics Committee (permit No. IACUC-20230928-01).

### 2.2. Experimental Design and Salinity Treatment

**Figure 1:**
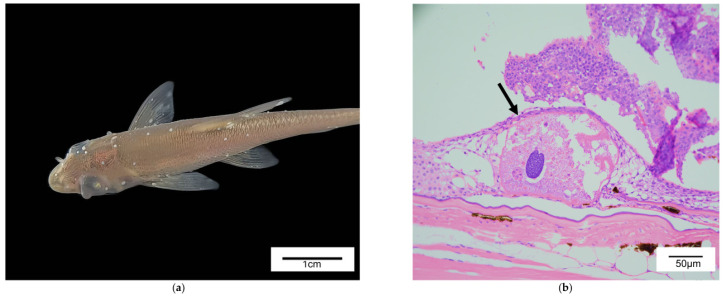
Morphological and histopathological characterization of *R. ventralis* with external symptoms: (**a**) External view of a representative fish exhibiting white spots on the body surface. (**b**) Skin histology (H&E staining, 40×) of the individual in (**a**), showing trophonts consistent with *I. multifiliis* in the epidermal layer (indicated by black arrows).

**Figure 2:**
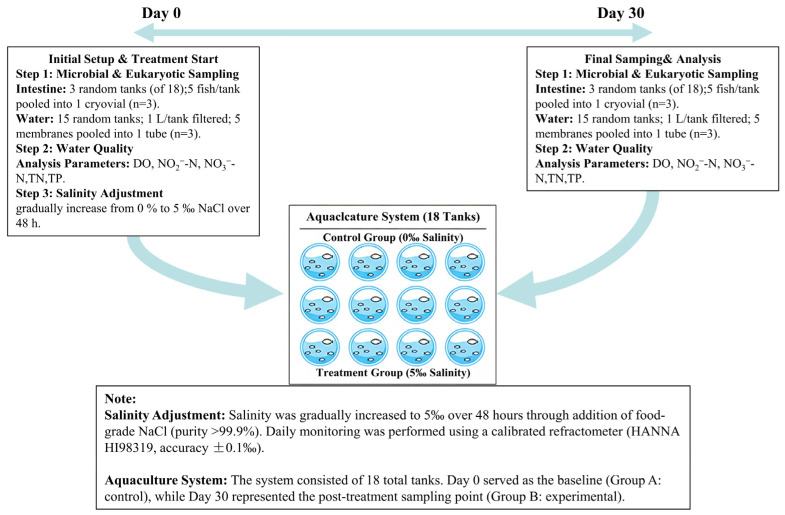
The schematic of the experiment design and timeline.

**Figure A1:**
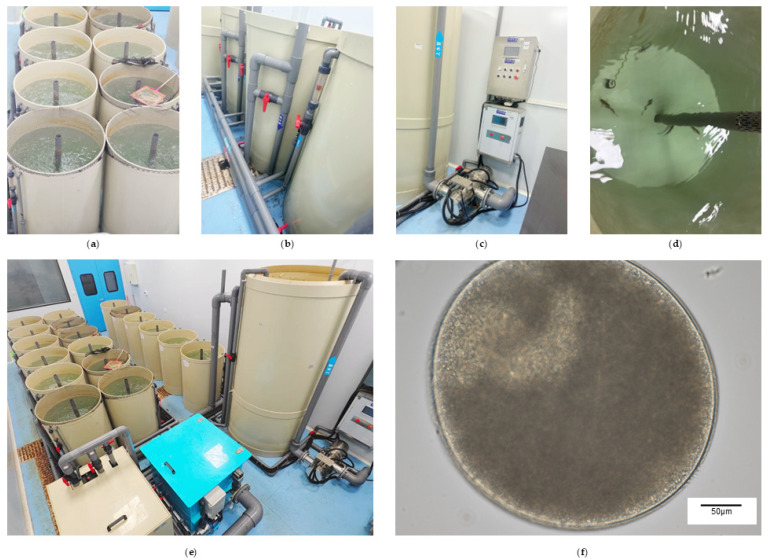
(**a**) Top view of multiple culture tanks. (**b**) Detailed view of the tank side plumbing, including water inlet, return pipes, and drainage valves used for water quality management. (**c**) Close-up of the main control panel and filtration unit of the RAS, highlighting the plumbing for water circulation. (**d**) Top-down view of the tank interior, showing the central water column and water conditions. (**e**) Overall view of the high-sterilization, high-filtration recirculating aquaculture system (RAS) room. (**f**) Microscopic image of an isolated trophont of *I. multifiliis*, morphologically characterized after collection from water samples.

We cultured *R. ventralis* sourced from a farm in Jingzhou, Hubei Province, in a high-sterilization, high-filtration recirculating aquaculture system (RAS) at the Integrative Science Center of Germplasm Creation in Western China (Chongqing, China) Science City (Figure A1). The culture tanks measured 0.8 m in diameter and 1.0 m in height, with a stocking density set at 50 fish per tank. Two weeks after culture initiation, the fish (average body length 5.14 ± 0.58 cm, average body weight 2.26 ± 0.19 g) exhibited visible signs, including a low density of white spots on the body surface (Figure 1a). Based on these characteristic signs, the condition was tentatively diagnosed as Ichthyophthiriasis [15]. Critically, skin histopathological examination (H&E staining, ×40) was performed on a representative individual to confirm the presence of *I. multifiliis* trophonts in the epidermal layer (Figure 1b). Since increasing water salinity to 5‰ is widely utilized in aquaculture to inhibit the free-living stages of aquatic eukaryotes, understanding its broader ecological consequences is crucial. Therefore, this preliminary study was designed to evaluate the ecological implications and potential trade-offs of salinity application at 5‰ by assessing its impact on the water prokaryotic/eukaryotic molecular communities and host intestinal microbial communities in *R. ventralis*. We employed an experimental intervention by increasing the water salinity to 5‰ and conducted a 30-day monitoring assay to observe the capacity of salinity to reduce the environmental molecular signal of *I. multifiliis* in the freshwater system. Our integrated assessment simultaneously monitored the water microbial and eukaryotic communities (16S and 18S rRNA), the fish intestinal microbiome (16S rRNA), and key water quality parameters. The baseline for this intervention was set as the day before salinity application commenced (Day 0). The experimental design and sampling timeline are illustrated in Figure 2.

During the trial, water salinity in treatment groups was maintained at 5.0 ± 0.3‰ using a portable salinity meter (HANNA HI98319; ±0.1‰ accuracy) by supplementing aerated tap water or NaCl solution (5‰). All tanks maintained a temperature of 22.0 ± 0.5 °C. Fish were fed commercial feed (3% of total biomass) twice daily at 09:00 and 17:00.

### 2.3. Sampling Protocols

#### 2.3.1. Intestinal Microbial Sampling

Sampling was conducted on Day 0 (pre-experiment) and Day 30 (after treatment with 5‰ salinity). Three tanks were randomly selected from 18 aquaculture tanks using a random number table. Five *R. ventralis* individuals were collected per selected tank, and their intestinal contents were aseptically dissected. Contents from five fish within the same tank were pooled into a sterile cryovial (1 vial/tank; 3 vials total), flash-frozen in liquid nitrogen, and stored at −80 °C for 16S rRNA sequencing.

#### 2.3.2. Water Microbial and Eukaryotic Community Sampling

Sampling was conducted on Day 0 and Day 30. Fifteen tanks were randomly selected, and 1 L of water was collected from each using sterile glass bottles. Each liter was vacuum-filtered through a 0.45 μm cellulose acetate membrane (Millipore, Burlington, MA, USA), yielding 15 membranes. Five membranes were randomly pooled into one 50 mL centrifuge tube (3 tubes total) and stored at −80 °C for 16S and 18S rRNA sequencing. The 18S rRNA sequencing data were used to quantify the relative abundance of *I. multifiliis* within the aquatic environment’s eukaryotic community, representing the circulating parasite load in the system. This approach provides a measure of molecular signal based on sequence proportions, which is not equivalent to classical parasitological metrics (e.g., mean abundance, prevalence, or intensity) proposed by Bush et al. [16]. Consequently, this molecular data should be interpreted solely as a description of changes in the environmental microbial composition.

#### 2.3.3. Water Quality Analysis

On Day 0 and Day 30, water samples were collected from three randomly selected tanks. On-site measurements of pH were taken using a portable pH meter (HANNA HI9811-5, Woonsocket, RI, USA; ±0.01 accuracy). Concentrations of dissolved oxygen (DO), nitrite nitrogen (NO_2_^−^-N), nitrate nitrogen (NO_3_^−^-N), total nitrogen (TN), and total phosphorus (TP) were measured within 24 h using a SEAL AA3 AutoAnalyzer (Merck KGaA, Darmstadt, Germany), following the Standard Methods for the Examination of Water and Wastewater (APHA, 23rd edition) [17].

### 2.4. Bacterial 16S rRNA Target Amplification and PCR Purification

Genomic DNA was extracted using the TIANGEN Genomic DNA Kit (TIANGEN, Beijing, China) and quantified via NanoDrop 2000 spectrophotometer (Thermo Fisher Scientific, Waltham, MA, USA). The V3-V4 region of bacterial 16S rRNA was amplified with primers 338F (5′-ACTCCTACGGGAGGCAGCAG-3′) and 806R (5′-GGACTACHVGGGTWTCTAAT-3′). PCR reactions (20 μL total) included 10 μL 2× Pro Taq DNA Polymerase Mix (Accurate Biotechnology, Changsha, China), 1 μL template DNA (10 ng/μL), 0.8 μL each primer (5 μM), and ddH_2_O. Cycling conditions: 95 °C for 3 min; 29 cycles of 95 °C/30 s, 53 °C/30 s, 72 °C/45 s; final extension at 72 °C/10 min. Amplicons were electrophoresed on 2% agarose gels (GelRed, Biotium, Fremont, CA, USA), purified using an Axygen Gel Extraction Kit (Axygen, Union City, CA, USA), and sequenced on Illumina NovaSeq 6000 (PE250 mode) by Majorbio Co., Ltd. (Shanghai, China).

### 2.5. Eukaryotic 18S rRNA Target Amplification and PCR Purification

The V9 region of eukaryotic 18S rRNA was amplified with primers Euk1391F (5′-GTACACACCGCCCGTC-3′) and EukBR (5′-TGATCCTTCTGCAGGTTCACCTAC-3′). Reaction conditions mirrored Section 2.4. Purified products were sequenced as above.

### 2.6. Data Processing

Microbial community analysis was performed using high-throughput sequencing on an Illumina NovaSeq 6000 platform (PE250 mode). Water microbiota were characterized via amplicon sequencing targeting the 16S rRNA gene (V3-V4 region, primers 338F/806R) and the 18S rRNA gene (V9 region, primers Euk1391F/EukBR), while intestinal microbiota were assessed using 16S rRNA gene sequencing. Raw sequences underwent quality control using Fastp (v0.23.2) [18], followed by denoising and generation of amplicon sequence variants (ASVs) with DADA2 (v1.26.0) [19]. ASVs were subsequently clustered into operational taxonomic units (OTUs) at 97% similarity. This approach was employed to facilitate direct comparison with foundational literature and established databases that utilize an OTU-based framework. Taxonomic classification of bacterial sequences utilized the SILVA database (v138), and eukaryotic sequences were classified using the PR2 database (v5.0). The relative abundance of *I. multifiliis* was determined by the proportion of its assigned 18S rRNA sequences within the total eukaryotic reads for each water sample. α-Diversity indices (Shannon, Simpson, ACE, Chao1) and β-diversity analysis (principal coordinates analysis (PCoA) based on Bray–Curtis dissimilarity) were conducted in QIIME2 (v2022.2) [20]. Significant differences in community composition were assessed using PERMANOVA (Adonis algorithm, 999 permutations) and ANOSIM; biomarker taxa were identified via LEfSe analysis (LDA score > 4, *p* < 0.05). Associations between microbial communities and environmental factors were evaluated using redundancy analysis (RDA) and Mantel tests (Pearson correlation, 999 permutations). Microbial co-occurrence networks were constructed based on SparCC correlations (|ρ| > 0.6, *p* < 0.01). This algorithm was specifically chosen because it is designed to infer true correlation structures within compositional data, such as microbial relative abundances, thereby avoiding the spurious correlations that can arise from standard methods like Spearman or Pearson correlation. Node topological properties were visualized in Gephi (v0.10.1).

## 3. Results

### 3.1. Water Microbial and Eukaryotic Molecular Signal Dynamics

#### 3.1.1. Microbial and Eukaryotic Diversity Response to Salinity

**Figure 3:**
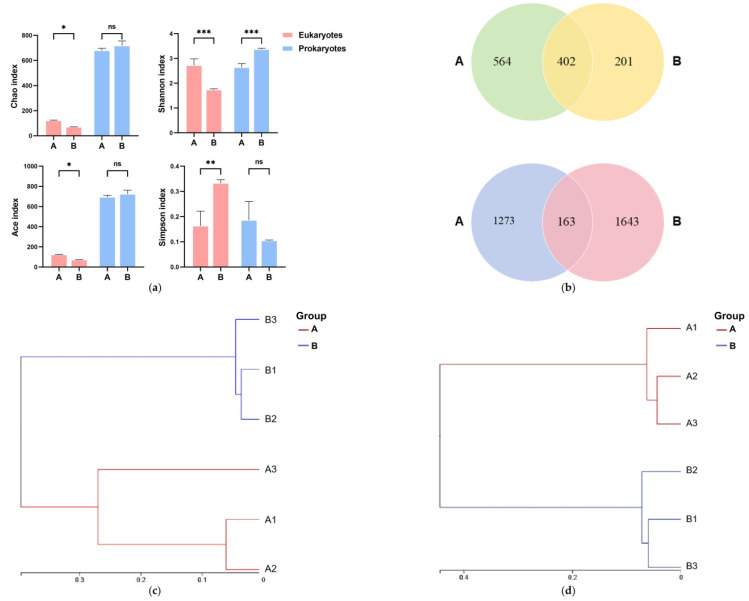
(**a**) Prokaryotic/eukaryotic α-diversity indices. (**b**) Venn diagram: prokaryotic (**top**)/eukaryotic (**bottom**) in water. (**c**) OTU-level clustering. (**d**) ASV-level clustering. Note: Statistical significance is indicated as follows: ns, not significant (*p* > 0.05); *, *p* < 0.05; **, *p* < 0.01; ***, *p* < 0.001.

After normalization, 358,978 prokaryotic and 607,235 eukaryotic high-quality sequences were retained (Coverage > 0.99; Table 1). For prokaryotes (Figure 3a), 5‰ salinity (Group B) significantly increased Shannon diversity (*p* < 0.05), indicating increased diversity, but did not alter ACE or Chao1 indices (*p* > 0.05). Eukaryotes exhibited reduced ACE, Chao1, and Shannon indices (*p* < 0.05) and an elevated Simpson index (*p* < 0.05), indicating suppressed richness and diversity under salinity application. Venn analysis (Figure 3b) revealed 1167 eukaryotic OTUs (Group A: 564 unique; Group B: 201 unique; shared: 402). Prokaryotic ASVs totaled 3079 (Group A: 1273 unique; Group B: 1643 unique; shared: 163 [5.29%]). UPGMA clustering (Figure 3c,d) showed high similarity within salinity groups (A1–A3; B1–B3), confirming salinity as a key driver of community divergence.

#### 3.1.2. Compositional Shifts in Water Microbial and Eukaryotic Communities

**Figure 4:**
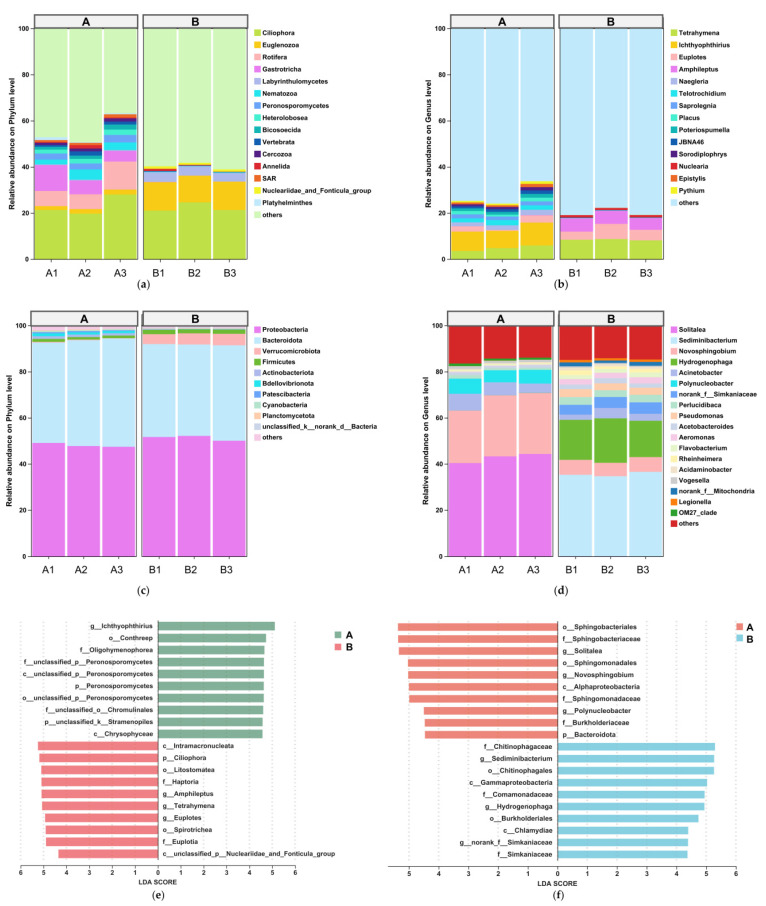
Microbial composition and biomarkers in water: (**a**) Eukaryotic phylum level. (**b**) Eukaryotic genus level. (**c**) Prokaryotic phylum level. (**d**) Prokaryotic genus level. (**e**) LEfSe: eukaryotic biomarkers (LDA > 4). (**f**) LEfSe: prokaryotic biomarkers (LDA > 4).

For eukaryotes, dominant phyla (relative abundance ≥ 1%) shared between groups at the phylum level (Figure 4a) included Ciliophora, Euglenozoa, Rotifera, and Gastrotricha. At the genus level (Figure 4b), shared dominant genera (≥1%) were *Ichthyophthirius*, *Amphileptus*, and *Saprolegnia*. For prokaryotes, dominant phyla (Figure 4c) across both groups were Proteobacteria, Bacteroidota, and Firmicutes, while shared dominant genera (≥1%) included *Solitalea*, *Sediminibacterium*, *Novosphingobium*, and *Hydrogenophaga* (Figure 4d).

LEfSe analysis (LDA score > 4; Figure 4e,f) revealed taxonomic differences between groups. In eukaryotes, Group A exhibited significant enrichment of *Ichthyophthirius*, whereas Group B showed higher abundances of *Amphileptus* and Euglenozoa. For prokaryotes, salinity treatment significantly reduced the relative abundance of *Solitalea*, *Novosphingobium*, Bacteroidota, *Polynucleobacter*, Sphingobacteriales, and Alphaproteobacteria. Conversely, taxa such as Sediminibacterium, Hydrogenophaga, Simkaniaceae, and Comamonadaceae, among others, were markedly enriched under elevated salinity. All reported differences were statistically significant (*p* < 0.05).

**Figure 5:**
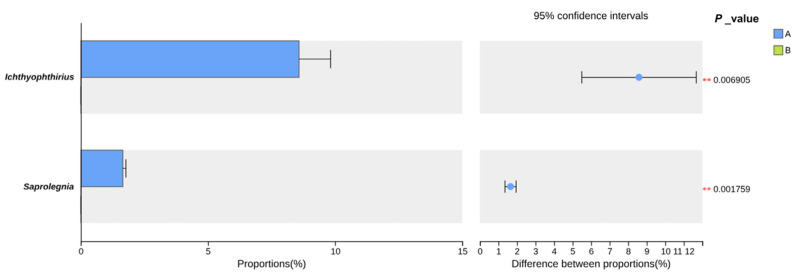
Relative Abundance of *Ichthyophthirius* and *Saprolegnia* before and after 5‰ salinity application. Note: ** indicates a highly significant difference (*p* < 0.01).

Results revealed a marked reduction in the molecular signal of key eukaryotic taxa in the aquaculture water following salinity application (Figure 5). The relative abundance of *Ichthyophthirius*, the causative agent of white spot disease, decreased from an average of 8.58% in the pre-treatment group (Group A) to undetectable levels in the post-treatment group (Group B) (*p* < 0.01). Similarly, the relative abundance of the oomycete *Saprolegnia* declined from an average of 1.65% in Group A to undetectable levels in Group B (*p* < 0.01).

#### 3.1.3. Effects of Salinity on Water Physicochemical Parameters

**Figure 6:**
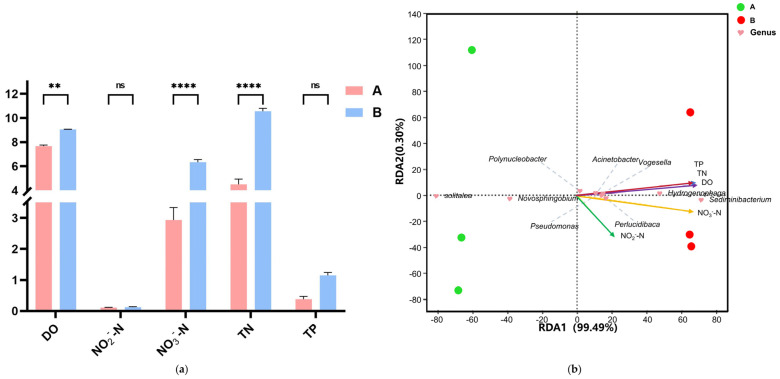
(**a**) Water quality parameters (DO, NO_3_^−^-N, TN, and TP) across salinity gradients. (**b**) RDA ordination plot showing physicochemical drivers of microbial communities. Note: Statistical significance is indicated as follows: ns, not significant (*p* > 0.05); **, *p* < 0.01; ****, *p* <0.0001.

Water quality parameters in the aquaculture system were analyzed (Figure 6a). Dissolved oxygen (DO), nitrate–nitrogen (NO_3_^−^-N), and total nitrogen (TN) concentrations were significantly higher in Group B than in Group A. Redundancy analysis (RDA) identified key environmental drivers of microbial community variation. RDA1 and RDA2 explained 99.49% and 0.30% of the variance in the top 10 genera, respectively (Figure 6b). NO_3_^−^-N, TN, total phosphorus (TP), and DO significantly influenced microbial community composition (*p* < 0.05). RDA1 was primarily driven by NO_3_^−^-N, TN, TP, and DO, with Group A samples negatively correlated and Group B samples positively correlated with RDA1.

### 3.2. Compositional Shifts in Intestinal Microbial Communities

**Figure 7:**
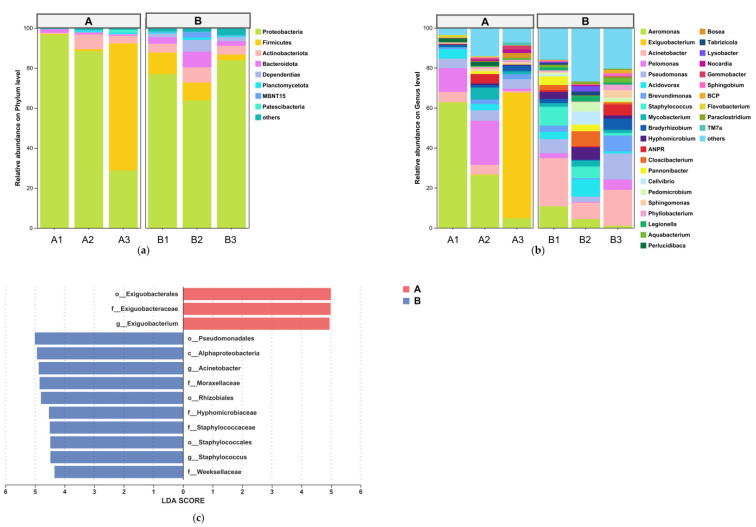
Intestinal microbiota dynamics: (**a**) Phylum composition. (**b**) Genus composition. (**c**) LEfSe biomarkers (LDA > 4).

At the phylum level (Figure 7a), the top 10 dominant phyla (relative abundance ≥1%) shared between groups included Proteobacteria, Firmicutes, Actinobacteriota, and Bacteroidota. At the genus level (Figure 7b), shared dominant genera (≥1%) among the top 10 taxa were *Aeromonas*, *Acinetobacter*, *Pelomonas*, and *Acidovorax*. LEfSe analysis (LDA score > 4; Figure 7c) revealed differential taxa from phylum to genus levels: Exiguobacterales, *Exiguobacterium*, and Exiguobacteraceae were significantly enriched in Group A, while Pseudomonadales, Alphaproteobacteria, *Acinetobacter*, Moraxellaceae, Rhizobiales, Hyphomicrobiaceae, *Hyphomicrobium*, *Staphylococcus*, *Cloacibacterium*, Weeksellaceae, Bacteroidia, Bacteroidota, and *Pedomicrobium* were enriched in Group B.

### 3.3. Water–Intestinal Microbial Interaction Networks

#### 3.3.1. Community Association Patterns

**Figure 8:**
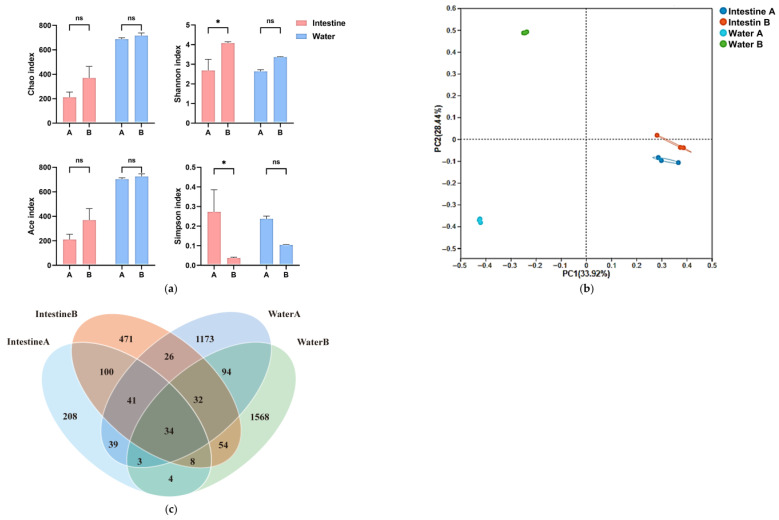
Cross-domain microbial interactions: (**a**) α-Diversity (water vs. intestinal). (**b**) PCoA ordination of water and intestinal communities. (**c**) Shared/unique OTUs between water and intestinal ecosystems. Note: Statistical significance is indicated as follows: ns, not significant (*p* > 0.05); *, *p* < 0.05.

Under 5‰ salinity treatment, increased Shannon index and decreased Simpson index (*p* < 0.05) in intestinal microbiota indicated enhanced species richness and evenness of intestinal microorganisms (Figure 8a). Significant spatial separation of communities confirmed by PCoA and ANOSIM (*p* = 0.001), combined with low water–intestinal shared OTU proportions (8.8–20.1%; 117/1645 vs. 128/2307) (Figure 8b,c), suggested that salinity influenced the structural characteristics of the intestinal microbial community. Although elevated salinity increased water OTU numbers, ACE/Chao indices consistently showed significantly higher microbial diversity in water than intestines (*p* < 0.05) without significant inter-group fluctuations, indicating greater stability of water microbiota against environmental disturbance compared to host-associated microbial communities.

#### 3.3.2. Key Species Interactions in Water and Intestinal Microbiota

**Figure 9:**
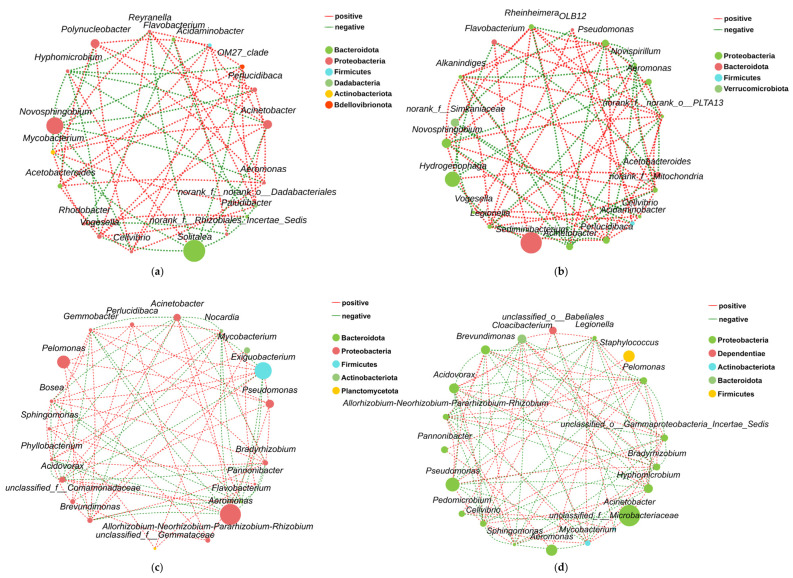
Microbial co-occurrence networks (Spearman’s |ρ| > 0.6, *p* < 0.05): (**a**) Water control group. (**b**) Water 5‰ salinity group. (**c**) Intestinal control group. (**d**) Intestinal 5‰ salinity group. Note: The size of each node corresponds to its degree (the number of connections with other nodes). The edges indicate the relationships between genera, with red lines representing positive connections and green lines representing negative connections.

Elevated salinity conditions induced divergent structural responses in the microbial co-occurrence networks of the intestine and aquatic environments (Figure 9). In the intestine, the network simplified, with a reduction in total edges (61 to 54) and a corresponding decrease in average degree (6.42 to 5.68), indicating lower connectivity (Figure 9c,d; Table 2). This was accompanied by a fundamental shift from a cooperative to a competitive dynamic, as the positive-to-negative edge ratio inverted from 1.90 to 0.93 (Table 2). This structural shift was marked by a succession of keystone phyla, where the control group’s core module, including Firmicutes, was replaced by a new module led by extremophiles like Deinococcota under elevated salinity conditions.

Conversely, the aquatic network became more complex and cooperative, with an increase in total edges (56 to 66) and average degree (5.89 to 6.60) (Figure 9a,b; Table 2). Cooperative interactions were strengthened, evidenced by an increased positive-to-negative edge ratio from 1.55 to 2.00 (Table 2). Here, the keystone genus shifted from a Solitalea-led module to a larger, more interconnected module headed by the highly adaptive *Pseudomonas*.

## 4. Discussion

### 4.1. Structural Impact of 5‰ Salinity on Environmental and Host Microbiomes

Our results reveal that salinity acts as a potent environmental filter, driving divergent and distinct structural changes in the aquatic and intestinal microbial communities.

#### 4.1.1. Aquatic Ecological Response

Salinity acts as a powerful environmental filter, driving divergent responses between prokaryotic and eukaryotic communities in the aquaculture water. Our results showed that 5‰ salinity significantly increased prokaryotic Shannon diversity, while concurrently reducing eukaryotic richness and diversity. This suggests that low salinity selected for halotolerant bacteria, allowing them to occupy newly available niches [21,22] while simultaneously suppressing osmosensitive eukaryotes [23]. This restructuring was evident at the community level, where co-occurrence network analysis revealed a significant increase in complexity, connectivity, and cooperative interactions in the salinity-treated group (Table 2). This structural reinforcement indicates an adaptive response, where the prokaryotic community formed new synergistic relationships to create a more stable and resilient ecosystem under osmotic stress [24]. The succession of the keystone genus from Solitalea to the highly adaptive Pseudomonas further underscores a functional reorganization toward taxa better suited for the modified environment [25].

The application of salinity significantly reduced the molecular signal of *I. multifiliis* and *Saprolegnia* spp. in the water (Figure 5), which is consistent with the documented effect of salinity on these pathogens in freshwater systems. Specifically, salinity levels > 3‰ inhibit *I. multifiliis* tomont hatching [7], and levels > 1‰ not only inhibit *Saprolegnia* hyphal extension osmotically [26] but also directly impair the pathogenicity of Saprolegnia parasitica by suppressing mycelial growth, zoospore release, and protease activity [27].

#### 4.1.2. Water Quality Changes and Impairment of Biogeochemical Function

LEfSe analysis revealed a significant reduction in the abundance of *Solitalea*, an important autotrophic denitrifier [28], while taxa with denitrifying capabilities like *Hydrogenophaga* and the new keystone genus *Pseudomonas* were enriched [29]. This indicates a functional replacement within the nitrogen-cycling guild. However, the observed increases in total nitrogen (TN) and nitrate–nitrogen (NO_3_^−^-N) in the water column suggest that this new consortium was less efficient at nitrogen removal than the original community under the closed-system conditions of this study. The suppression of key functional taxa like *Solitalea* appears to have created a bottleneck in the denitrification process, leading to nutrient accumulation. This demonstrates that even when a microbial community adapts structurally, critical ecosystem functions like nutrient remediation can be impaired.

### 4.2. Salinity Impacts on Intestinal Microbial Consortia

#### 4.2.1. Salinity-Driven Modulation of Intestinal Microbial Diversity

Salinity elevation induces significant restructuring of fish intestinal microbial diversity and functional profiles. Our study revealed that 5‰ salinity substantially enhanced alpha diversity and operational taxonomic unit (OTU) richness in the intestinal microbiota of *R. ventralis*. This aligns with observations in *Pangasianodon hypophthalmus* juveniles exhibiting maximal alpha diversity under 5‰ salinity [30]. In stark contrast to the adaptive response observed in the water, salinity stress destabilized the intestinal microbiome. While α-diversity metrics like the Shannon index increased, this was not indicative of a healthier community. Instead, co-occurrence network analysis revealed a significant simplification of the intestinal network, with fewer connections and a fundamental shift from cooperative to antagonistic interactions (Table 2). A loss of network stability and a rise in negative correlations are hallmarks of a disturbed and dysbiotic microbial community [31], which is a key indicator of compromised host health and an associated risk factor for increased susceptibility to secondary infections and disease in aquaculture [32,33]. This dysbiosis was characterized by a major shift in core phyla, where the keystone module led by Firmicutes was displaced by one dominated by extremophilic phyla like Deinococcota. This suggests that the increase in species richness was likely due to the breakdown of the stable, resident community, allowing transient or opportunistic taxa to colonize, rather than an enhancement of ecosystem function.

#### 4.2.2. Enrichment of Potentially Opportunistic Taxa in the Intestinal Microbiota

Salinity-associated shifts in the intestinal microbiota of *R. ventralis* revealed significant enrichment of opportunistic pathogens under 5‰ salinity, including Staphylococcus, Bacteroidota, Moraxellaceae, Weeksellaceae, *Acinetobacter*, and Pseudomonadales. Staphylococcus members produce enterotoxins implicated in mortality events in tilapia and mink [34,35]. Moraxellaceae encompasses facultative pathogens associated with visceral colonization, metabolic dysregulation, growth retardation, and immunosuppression in fish [36]. Bacteroidota-derived lipopolysaccharides (LPSs) induce inflammatory responses and intestinal barrier dysfunction [37], while Weeksellaceae exacerbates intestinal inflammation and disrupts villus morphogenesis [38]. *Acinetobacter* species, notably *A. junii*, provoke intestinal pathologies [39,40], and *Pseudomonas* members are established etiological agents of hemorrhagic septicemia, ulceration, ascites, and hepatic lesions in fish [41]. This increase in potentially opportunistic microorganisms reflects a significant structural shift in the intestinal microbiota.

#### 4.2.3. Impact of Salinity on Potential Beneficial Taxa

The 5‰ salinity treatment resulted in a significant reduction in *Exiguobacterium* abundance within the intestinal microbiota of cultured fish. As a potential probiotic genus, *Exiguobacterium* members synthesize amylases, lipases, and proteases to enhance decomposition of complex nutrients in feedstuffs, while fermenting carbohydrates to produce short-chain fatty acids (SCFAs) like propionate and butyrate, thereby improving nutrient absorption efficiency and reinforcing intestinal epithelial barrier function [42]. This genus may further inhibit pathogen proliferation through nutrient competition or antimicrobial compound secretion [43]. The salinity-induced reduction in probiotic abundance suggests that interventions such as probiotic supplementation warrant further investigation to maintain intestinal homeostasis when modulating the aquatic environment with salinity.

#### 4.2.4. Salinity-Driven Aquatic-Intestinal Microbial Cross-Domain Interactions

The opposing responses of the intestinal and water microbial networks to salinity stress highlight fundamentally different ecological strategies in host-constrained versus open environments. The simplification and shift to antagonism in the intestinal network are characteristic of dysbiosis, where environmental stress destabilizes the community by intensifying internal competition for resources, ultimately compromising host health [31]. The emergence of Deinococcota, a phylum known for extreme stress resistance [44], signifies an adaptive response at the microbial level but likely at the expense of beneficial host-symbiont functions [45]. In contrast, the aquatic microbial community adapted by enhancing network complexity and cooperation. This structural reinforcement suggests an adaptive strategy where microbes form new synergistic relationships to collectively overcome the environmental challenge, leading to a more resilient and stable ecosystem [46]. The succession to a Pseudomonas-dominated core reflects a functional reorganization toward genera better equipped to thrive under saline conditions [25]. For aquaculture, these findings underscore a critical duality: while the external environment’s microbiota may adapt robustly, the same stressor can destabilize the internal microbiome crucial for fish health, necessitating management strategies that consider both ecosystems [14].

Overall, this preliminary assessment reveals a key ecological trade-off: the significant reduction in the environmental molecular signal of *I. multifiliis* and *Saprolegnia* spp., at 5‰ salinity, coincided with marked destabilization of the intestinal microecology. In this context, supplementation with probiotic genera, such as *Bacillus* and *Lactobacillus*, represents a promising avenue for future research [47,48]. These probiotics are known to improve intestinal health by competitively excluding opportunistic pathogens, producing antimicrobial compounds, and modulating host immunity, mechanisms that could potentially help restore microbial balance and enhance disease resistance in fish [49].

## 5. Conclusions

While the application of 5‰ salinity successfully reduced the molecular signal of key pathogens (*I. multifiliis* and *Saprolegnia* spp.) in the water, this study reveals that this reduction in pathogen molecular signals is accompanied by previously overlooked costs to host health and ecosystem function. The treatment induced a systemic restructuring of microbial communities, characterized by several critical shifts:

(1) In the aquatic environment, increased prokaryotic diversity and network complexity coincided with impaired self-purification capacity, evidenced by elevated dissolved oxygen, nitrate nitrogen, and total nitrogen.
(2) In the host, the intestinal microbiome underwent significant destabilization; this was characterized by a simplification of its interaction network, a surge in potentially opportunistic taxa, and a decline in potential beneficial probiotics, all collectively indicating a state of dysbiosis.
(3) A fundamental divergence in microbial adaptation strategies was observed: the environmental community enhanced its resilience through increased cooperation, whereas the host-associated intestinal community shifted toward a stressed, antagonistic state.

These findings underscore a critical ecological trade-off: the established practice of using salinity for pathogen control achieves its goal at the direct expense of host microbiome integrity and aquatic ecosystem functioning. Therefore, moving forward, aquaculture management must look beyond merely suppressing waterborne pathogens and adopt an integrated perspective that equally prioritizes host internal health and the stability of the water microbiome. Future strategies should explore combining salinity application with microbiome-supporting interventions, such as targeted probiotic supplementation, to mitigate these adverse effects and foster a more sustainable, health-oriented aquaculture system.

## Figures and Tables

**Table 1:** Summary of high-throughput sequencing data (reads, OTUs, ASVs, coverage).

Sample ID	Prokaryotes			Eukaryotes		
	Sequences (×10^3^)	ASV Number	Coverage	Sequences (×10^3^)	OTU Number	Coverage
A1	53.64	690	0.998446	99.64	741	0.999909
A2	52.72	654	0.998446	97.24	711	0.999909
A3	59.14	656	0.997579	88.14	673	0.999943
B1	60.76	700	0.998356	95.63	459	0.999897
B2	52.28	685	0.999863	99.09	454	0.99992
B3	55.24	759	0.999641	104.07	469	0.99992

**Table 2:** Topological properties of microbial co-occurrence networks in the intestine and water microbiota under control and salinity application.

Parameter	Intestine Control	Intestine Salinity	Water Control	Water Salinity
Nodes	19	19	19	20
Edges	61	54	56	66
Positive Edges	40	26	34	44
Negative Edges	21	28	22	22
Positive/Negative Ratio	1.9	0.93	1.55	2
Average Degree	6.42	5.68	5.89	6.6
Avg. Clustering Coefficient	0.62	0.79	0.58	0.61

Note: Networks were constructed based on Spearman correlation (|*r*| > 0.6, *p* < 0.05).

## Data Availability

The original contributions presented in this study are included in the article. Further inquiries can be directed to the corresponding authors.

## Abbreviations

The following abbreviations are used in this manuscript:

*R. ventralis*	*Rhinogobio ventralis*
*I. multifiliis*	*Ichthyophthirius multifiliis*
RAS	Recirculating aquaculture system
OTUs	Operational taxonomic units
ASVs	Amplicon sequence variants
RDA	Redundancy analysis
PCoA	Principal co-ordinates analysis
DO	Dissolved oxygen
NO_3_^−^-N	Nitrate nitrogen
TN	Total nitrogen
